# YOLO-Night: Lighting the Path for Autonomous Vehicles with Robust Nighttime Perception

**DOI:** 10.3390/s26041138

**Published:** 2026-02-10

**Authors:** Jinxin Tian, Muhammad Arslan Ghaffar, Zhaokai Li

**Affiliations:** 1 Henan Mechanical and Electrical Vocational College, Zhengzhou 451191, China; 2 Shenzhen Institute of Advanced Technology, Chinese Academy of Sciences, Shenzhen 518055, China; 3 University of Chinese Academy of Sciences, Beijing 100049, China; 4 School of Automobile, Chang’an University, Xi’an 710064, China

**Keywords:** nighttime object detection, low-light perception, autonomous driving, YOLO-based detectors, multi-scale feature fusion, real-time inference

## Abstract

**Highlights:**

**What are the main findings?**

- A nighttime-oriented YOLO framework (YOLO-Night) is proposed, integrating feature conditioning, adaptive receptive fields, and staged multi-scale fusion to improve detection robustness under low-illumination conditions.
- YOLO-Night achieved substantially higher precision and mAP on the NightCity dataset than lightweight YOLO baselines and nighttime-oriented detectors while maintaining real-time inference with moderate computational overhead.

**What are the implications of the main findings?**

- The results demonstrate that architectural adaptation within the detection pipeline is more effective than standalone image enhancement for nighttime autonomous driving perception.
- YOLO-Night provides a practical accuracy–efficiency trade-off suitable for real-world deployment in autonomous vehicles and Advanced Driver Assistance Systems (ADAS).

**Abstract:**

Despite substantial progress in visual perception, object detection systems for autonomous driving still exhibit pronounced performance degradation in nighttime and low-light conditions, where reduced signal-to-noise ratio, blurred object boundaries, and scale ambiguity challenge reliable recognition. Existing YOLO-based detectors, primarily optimized for daytime imagery, struggle to maintain robustness under such adverse illumination. To address these issues, we propose YOLO-Night, a nighttime-oriented object detection framework that enhances the YOLO11 architecture through a structured integration of contrast enhancement, adaptive receptive field modeling, and multi-scale feature fusion. The framework incorporates a feature-level enhancement mechanism to improve low-contrast representations, employs depthwise switchable atrous convolution to dynamically adapt receptive fields for blurred and small objects, and introduces a multi-scale convolutional block to strengthen feature extraction under severe illumination degradation. In addition, a staged feature fusion strategy with an auxiliary low-level detection head was adopted to mitigate semantic misalignment across feature scales. Extensive experiments on the NightCity dataset demonstrated that YOLO-Night consistently outperformed the YOLO11n baseline, achieving improvements of +14.3% precision, +12.4% recall, and +10.4% mAP@50 under nighttime conditions while maintaining real-time inference capability. These results indicate that targeted architectural adaptations can substantially improve object detection robustness in low-light driving scenarios.

## 1. Introduction

Object detection is a core component of autonomous driving perception systems [1], enabling reliable scene understanding for tasks such as obstacle avoidance [2,3], trajectory planning [4], and decision-making [5]. While modern deep learning-based detectors have achieved remarkable performance under favorable illumination, their reliability degrades substantially in nighttime and low-light driving scenarios. In such conditions, reduced signal-to-noise ratios, blurred object boundaries, and diminished inter-class contrast fundamentally compromise visual feature representations, leading to missed detections and unstable predictions [6]. Nighttime perception presents challenges that differ qualitatively from those encountered in daytime environments [7]. First, extremely low illumination causes a collapse of discriminative visual cues, where conventional convolutional features fail to preserve object structure under severe noise and contrast degradation [8]. Second, dense nighttime traffic scenes exacerbate scale ambiguity and partial occlusion, particularly for small or distant objects whose features are easily suppressed during multi-scale aggregation. Third, autonomous driving systems impose strict real-time constraints, requiring detectors to maintain high inference speed while preserving robustness under adverse illumination—a balance that remains difficult for existing architectures optimized primarily for daytime imagery [9].

Recent YOLO-based detectors emphasize lightweight design and efficient feature pyramids; however, they typically rely on fixed receptive fields and generic feature fusion strategies that are insufficiently adaptive to nighttime-specific degradation. Image enhancement methods and multi-scale detection strategies have been explored independently, yet their integration into a unified real-time detection framework for nighttime driving remains underexplored. In particular, the interaction between contrast enhancement, receptive field adaptation, and cross-scale feature alignment has not been systematically addressed in existing YOLO-style pipelines.

To address these limitations, we propose YOLO-Night, a nighttime-oriented object detection framework built upon the YOLO11 architecture. The framework introduces a structured combination of feature enhancement [10], adaptive receptive field modeling [11], and staged multi-scale feature fusion [12] to improve robustness under low-light conditions. Specifically, feature-level contrast enhancement is employed to mitigate representation degradation in extremely dark scenes, adaptive atrous convolution is used to accommodate scale variation and blur, and an enhanced multi-scale fusion strategy with a low-level detection head is adopted to reduce semantic misalignment across feature hierarchies [13]. Extensive experiments on the NightCity dataset demonstrate that the proposed framework consistently outperforms the YOLO11n baseline in nighttime detection accuracy while preserving real-time inference capability.

## 2. Preliminary

### 2.1. Related Work

Early object detection methods relied on handcrafted features such as Histogram of Oriented Gradients (HOG) [14] and Scale-Invariant Feature Transform (SIFT) [15], which demonstrated reasonable performance under well-illuminated and high-quality imaging conditions. However, these methods are highly sensitive to illumination changes, noise, and contrast degradation, making them unsuitable for complex nighttime or low-light environments. With the advancement of deep learning, Convolutional Neural Network (CNN)-based detectors, including Faster R-CNN [16], SSD [17], and the YOLO series [18], have significantly improved detection accuracy and robustness through end-to-end training. Among them, YOLO-based detectors achieve a favorable balance between accuracy and inference speed, making them widely adopted in real-time applications such as autonomous driving.

To address low-light conditions, existing nighttime object detection approaches often integrate image enhancement techniques with detection models. Representative enhancement methods include RetinexNet [19], EnlightenGAN [20], and Zero-DCE [21], which aim to restore visibility and contrast before detection. However, such preprocessing-based pipelines frequently suffer from noise amplification and increased computational overhead, limiting their effectiveness and deployment on resource-constrained platforms.

Several studies have explored nighttime detection without explicit pixel-level enhancement. Han et al. proposed 3L-YOLO [22], which employs multi-scale feature aggregation and dynamic detection heads to improve nighttime detection performance; however, its ability to detect small objects remains limited. Jiang et al. proposed LOL-YOLO by integrating SCINet [23] to balance computational efficiency and detection accuracy under low-light conditions [24]. While this approach reduces model complexity, its reliance on global enhancement limits robustness in extremely dark scenes, where aggressive illumination amplification can distort local textures and lead to unstable feature representations. As a result, the detection performance degrades when object boundaries are severely blurred or contrast is highly uneven. Peng et al. introduced NLE-YOLO [25], which combines low-frequency filtering with feature enhancement modules to improve detection accuracy in nighttime environments. Although effective in enhancing global visibility, the low-frequency emphasis may suppress fine-grained details critical for small or distant object detection. In addition, the introduction of multiple enhancement and filtering modules significantly increases computational complexity, constraining real-time deployment on edge devices commonly used in autonomous driving systems. Overall, despite recent progress, nighttime object detection remains challenging. Existing methods either rely heavily on image enhancement, which can amplify noise and increase inference cost [26], or struggle to maintain stable performance when confronted with extremely low contrast, blurred targets, and small objects. These limitations highlight the need for lightweight detection frameworks that enhance robustness under adverse illumination while preserving real-time performance.

### 2.2. Baseline Architecture

The YOLO family of detectors has undergone continuous evolution with a primary focus on achieving real-time performance while maintaining competitive detection accuracy. The original YOLO framework [18] reformulated object detection as a single-stage regression problem, significantly improving inference speed. Subsequent versions, including YOLOv2 [27] and YOLOv3 [28], enhanced detection accuracy through the introduction of anchor boxes and multi-scale feature representations. YOLOv4 [29] and YOLOv5 [30] further improved performance by incorporating advanced data augmentation strategies and architectural components such as CSPNet and PANet. Recent versions, from YOLOv6 to YOLOv11, emphasize efficiency, scalability, and adaptability across different computational budgets by leveraging neural architecture search, reparameterizable convolutions, and optimized feature extraction blocks. These developments have driven YOLO-based detectors toward higher accuracy, reduced model size, and improved inference efficiency, making them well-suited for real-time perception tasks.

**Figure 1:**
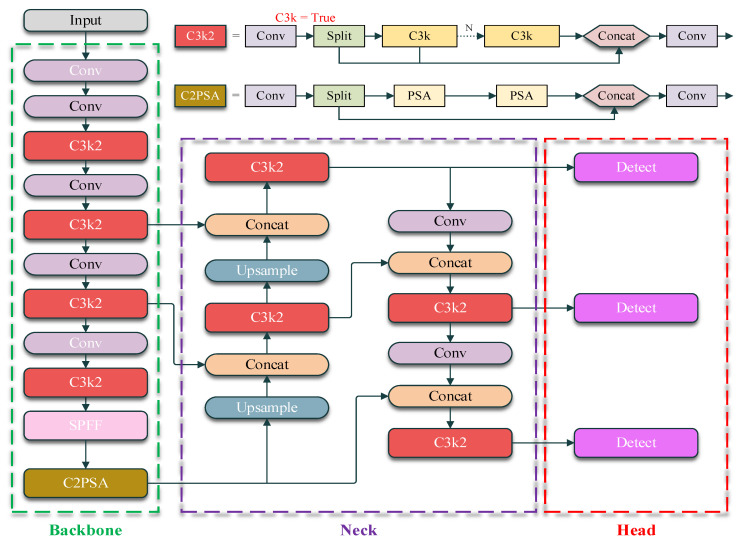
Network architecture of YOLO11 (baseline), consisting of a convolutional backbone, feature aggregation neck, and multi-scale detection head.

YOLO11 [31], released in October 2024, serves as the baseline architecture in this study, and its overall network structure is illustrated in Figure 1. Compared with YOLOv8, YOLO11 replaces the C2f module with C3k2 and introduces a Cross Stage Partial with Parallel Spatial Attention (C2PSA) module after the Spatial Pyramid Pooling Fast (SPPF) layer. The C2PSA module processes features through parallel partial spatial attention branches before fusion, enabling enhanced spatial feature representation. These architectural improvements allow YOLO11 to better preserve fine-grained information and enhance sensitivity to small or partially occluded objects, making it a suitable baseline for further adaptation to nighttime and low-light object detection tasks.

### 2.3. Key Limitations

Despite achieving competitive performance under normal illumination, YOLOv11n exhibits several critical limitations when deployed in low-light and nighttime environments, which hinder reliable perception in real-world scenarios. First, under extremely low illumination, the visual signal captured by RGB sensors suffers from severe noise contamination and contrast degradation, resulting in a substantial loss of discriminative information. When processed by convolutional backbones optimized for well-lit images, such degradation leads to unstable feature representations in deeper layers. In particular, noise patterns are often propagated through multi-scale feature fusion pathways, causing false activations that resemble object textures, confusion between dark object regions and background areas, and weakened edge cues that are essential for small-object detection, such as pedestrians and animals. Second, YOLOv11n treats RGB channels uniformly during feature extraction, despite the fact that different channels respond differently under low-light conditions. In nighttime imagery, useful structural information becomes unevenly distributed across channels, while noise components may dominate specific spectral responses. As a result, conventional convolutional operations amplify channel-wise noise indiscriminately, leading to degraded feature quality and reduced robustness in subsequent detection stages. Third, although YOLOv11n incorporates attention-based mechanisms to enhance spatial feature representation, these modules are primarily optimized for well-illuminated scenes. Under low-light conditions, attention responses tend to be influenced by high-frequency noise and illumination artifacts, particularly for distant or low-contrast objects and specular reflections, which can be mistakenly interpreted as distinct targets. This limits the effectiveness of attention in distinguishing semantically meaningful regions from background clutter at night. Finally, YOLOv11n performs frame-wise inference without exploiting temporal correlations inherent in video-based autonomous driving scenarios. In nighttime environments, sensor noise varies randomly across frames, whereas true objects exhibit temporal consistency. The absence of temporal modeling prevents the detector from leveraging motion coherence to suppress noise-induced false detections and stabilize predictions across consecutive frames.

These limitations collectively result in a substantial degradation of detection performance when YOLOv11n is evaluated on nighttime benchmarks such as NightCity and ExDark, underscoring the need for illumination-robust architectural adaptations rather than relying solely on data augmentation or daytime-oriented model designs.

## 3. YOLO-Night Algorithm

### 3.1. YOLO-Night Architecture

**Figure 2:**
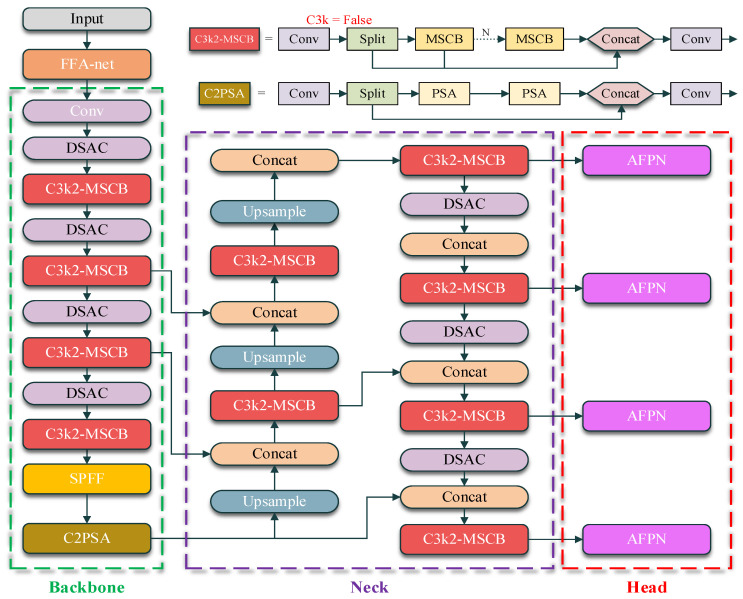
Overview of the YOLO-Night architecture for nighttime object detection. The network integrates feature enhancement, adaptive receptive field modeling, and multi-scale feature fusion within a unified YOLO-based backbone, neck, and detection head to improve robustness under low-light conditions.

The proposed YOLO-Night architecture is designed as a unified detection framework that explicitly addresses feature degradation caused by low illumination, noise, and scale ambiguity in nighttime environments. The overall network structure is illustrated in Figure 2. Rather than treating nighttime challenges independently, YOLO-Night adopts a staged design that enhances feature robustness, adapts receptive fields, and improves cross-scale feature alignment within a single real-time detection pipeline. At the input stage, a feature enhancement module is integrated to alleviate severe contrast degradation and stabilize feature representations extracted from low-light inputs. This enhancement is embedded directly into the detection pipeline to suppress illumination-induced noise before deep feature extraction. To improve robustness against blur and scale variation commonly observed in nighttime driving scenarios, adaptive receptive field modeling is incorporated within the backbone and neck. In parallel, multi-scale feature extraction is strengthened to preserve fine-grained details and mitigate feature suppression caused by partial occlusion and background interference. Finally, a staged feature fusion strategy with an additional low-level detection branch is employed in the detection head to reduce semantic misalignment across feature scales. This design enhances the detection of small and low-contrast objects in complex nighttime scenes.

Through the coordinated integration of feature enhancement, adaptive receptive field modeling, multi-scale feature extraction, and staged feature fusion, YOLO-Night forms a coherent architecture tailored for robust object detection in dark and low-light environments while maintaining real-time inference capability.

### 3.2. FFA-Net

The Feature Fusion Attention Network (FFA-Net) was originally designed for image dehazing [10], but its core strengths, channel attention (CA) and pixel attention (PA), are uniquely suited to address nighttime-specific contrast collapse and noise contamination. Unlike daytime scenes, low-light environments suffer from unevenly distributed structural information across RGB channels (e.g., noise dominates blue channels, while red/green channels retain faint object edges) and suppressed local textures (e.g., pedestrian clothing or vehicle contours blending into dark backgrounds).

FFA-Net’s CA mechanism targets channel-wise imbalance by adaptively reweighting feature responses (Equations (4) and (5)), amplifying channels carrying residual structural cues (edge information in low-noise channels) while suppressing noise-dominated channels. This is critical for nighttime scenes, where generic channel-agnostic convolutions indiscriminately propagate noise. The PA module further refines spatial features (Equations (6) and (7)) by focusing on locally informative regions (dimly lit vehicle taillights or pedestrian silhouettes) that are easily lost in low-contrast nighttime imagery. Unlike dehazing, where FFA-Net restores global visibility, its application here is tailored to preserve discriminative object features amid illumination-induced noise, a challenge distinct from haze (which uniformly scatters light).

**Figure 3:**
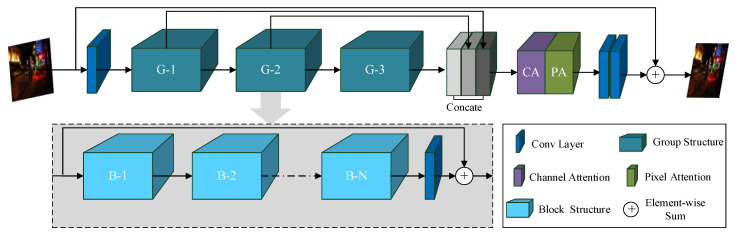
Architecture of the Feature Fusion Attention Network (FFA-Net), illustrating the integration of channel attention (CA) and pixel attention (PA) for adaptive feature refinement under low-contrast conditions.

By embedding FFA-Net at the early stage of the detection pipeline, we stabilize low-level feature representations before deep extraction, directly mitigating the “discriminative cue collapse” that plagues nighttime detection. The network structure of FFA-Net is shown in Figure 3.

As illustrated in Figure 3, CA captures global channel-wise statistics via global average pooling, generating channel descriptors:

(1) $$g_{c}=H_{p}(F_{c})=\frac{1}{H\times W}\sum_{i=1}^{H}\sum_{j=1}^{W}x_{c}(i,j)$$

where $x_{c}\;(i,j)$ represents the position of the *c*-th channel at (i,j), and $H_{p}$ represents the global pooling function.

The mathematical expression for generating channel attention weights is:

(2) $$W_{CA}=\sigma \left\{Conv\left\{\delta \left[Conv\left(g_{c}\right)\right]\right\}\right\}$$

where σ represents the Sigmoid function and δ represents the ReLU function. By multiplying the input feature ($F_{c}$) with the channel attention weights ($W_{CA}$), the channel-refined feature map ($F^{*}$) is obtained:

(3) $$F^{*}=W_{CAc}⊗F_{c}$$

here, $F^{*}$ represents the intermediate feature map after channel attention refinement, which serves as the input to the pixel attention (PA) module.

PA further refines spatial feature responses by focusing on locally informative regions through pixel-wise attention:

(4) $$F_{PA}=\sigma \left\{Conv\left\{\delta \left[Conv\left(F^{*}\right)\right]\right\}\right\}$$

multiply $F^{*}$ by $F_{PA}$ element by element:

(5) $$\tilde{F}=F^{*}⊗F_{PA}$$

**Figure 4:**
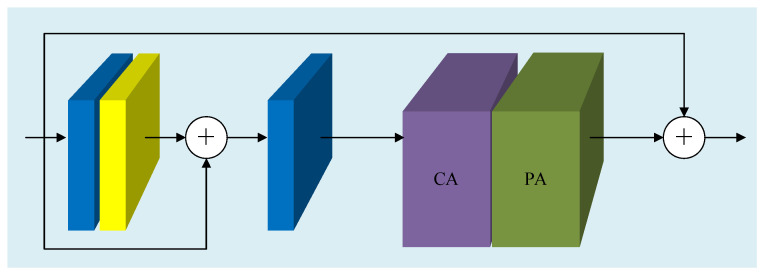
Basic residual block of FFA-Net with feature attention, combining channel and pixel attention mechanisms to enhance informative features while suppressing noise.

The basic building block of FFA-Net, shown in Figure 4, integrates residual learning with feature attention, enabling stable feature refinement through stacked modules. In the proposed YOLO-Night framework, FFA-Net is applied at the early stage to enhance low-level feature representations before deep feature extraction, providing more reliable inputs for subsequent detection under low-light conditions. FFA-Net uses a simple L1 loss as the default loss function, and its formula is as follows:

(6) $$L(\Theta )=\frac{1}{N}\sum_{i=1}^{N}\left\|I_{gt}^{i}-FFA(I_{in}^{i})\right\|$$

In the formula, Θ represents the parameters of FFA-Net, $I_{gt}$ represents real images, and $I_{in}$ represents the input image.

FFA-Net, through its introduction of channel attention and pixel attention mechanisms, effectively enhances the expression ability of key regions and detail features in images, and improves the perceptibility of targets in dark and low contrast environments.

### 3.3. DSAC

Nighttime driving images often suffer from motion-induced blur and severe detail loss caused by long exposure times and low signal-to-noise ratios. These effects make it difficult for fixed receptive field convolutions to simultaneously capture fine-grained details and broader contextual information. To address this issue, YOLO-Night incorporates Depthwise Switchable Atrous Convolution (DSAC) [11] to enable adaptive receptive field adjustment under low-light conditions.

**Figure 5:**
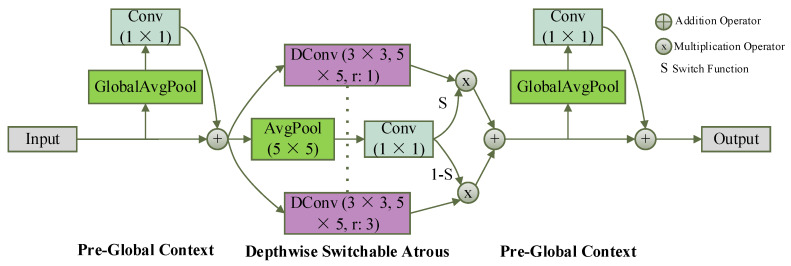
Architecture of Depthwise Switchable Atrous Convolution (DSAC), illustrating parallel depthwise convolutions with different dilation rates and a switch mechanism for adaptive receptive field selection.

Atrous (dilated) convolution has been widely adopted in detection and segmentation tasks to expand the receptive field without increasing kernel size [32,33,34]. However, using a single dilation rate is insufficient for handling the wide range of object scales and blur levels present in nighttime scenes. As illustrated in Figure 5, DSAC integrates global context modeling with parallel depthwise convolutions using different dilation rates and dynamically fuses their outputs through a switch mechanism. Unlike general convolution, DSAC is trained to prioritize nighttime-specific challenges, it uses standard convolution for small, low-contrast targets (e.g., distant pedestrians) and dilated convolution for blurred objects (e.g., moving vehicles in dim light), outperforming fixed receptive fields that fail to balance these low-light-specific tradeoffs.

The operation of DSAC can be expressed as:

(7) $$y=S(x)\cdot Conv(x,w,1)+\left[1-S(x)\right]\cdot Conv(x,w+\Delta w,r)$$

where x represents the input feature map, w is the pre-trained weight for standard convolution, Δw is a trainable weight offset term, and r is the set void ratio.

Here, S(x) is a learnable switch function implemented using global average pooling followed by a 1×1 convolution and a Sigmoid activation, producing continuous gating values in [0, 1]. The parameters of S(x) are trained end-to-end together with the network, and are used to dynamically control the fusion ratio of standard convolution and dilated convolution.

By jointly modeling local details and broader contextual cues, DSAC enables the network to adapt its receptive field according to target scale and blur severity. In YOLO-Night, DSAC is embedded within both the backbone and neck to enhance feature robustness for blurred and multi-scale targets while maintaining efficient computation.

### 3.4. C3k2-MSCB

To improve feature robustness under scale imbalance and partial feature suppression in low-light environments, the C3k2 module in YOLO11 is extended with a Multi-Scale Convolution Block (MSCB) [12], forming the proposed C3k2-MSCB. This design aims to enhance multi-scale feature representation while maintaining lightweight computation.

While MSCB was initially used for medical imaging, its adaptation here targets nighttime-specific feature suppression, as small objects (e.g., bicycles) and low-contrast targets lose fine details in dark scenes, and MSCB’s parallel kernels and residual connections preserve these cues better than generic multi-scale modules optimized for well-lit data. Smaller kernels focus on local structures and fine details, while larger kernels provide broader contextual cues. By aggregating these responses, the network improves its ability to preserve object features that may be weakened by low contrast, blur, or partial occlusion in nighttime scenes. To facilitate effective information fusion across scales, channel shuffle and residual connections are employed, promoting feature diversity and stabilizing gradient propagation. The resulting C3k2-MSCB module enhances semantic consistency across feature maps and mitigates the loss of discriminative information during deep feature extraction.

**Figure 6:**
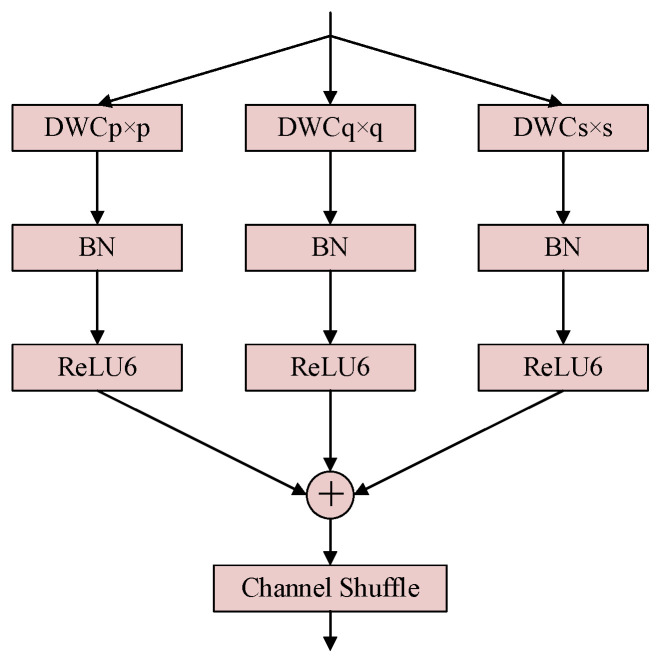
Structure of the multi-scale depthwise convolution (MSDC) module, employing parallel depthwise convolutions with different kernel sizes to capture multi-scale spatial features.

**Figure 7:**
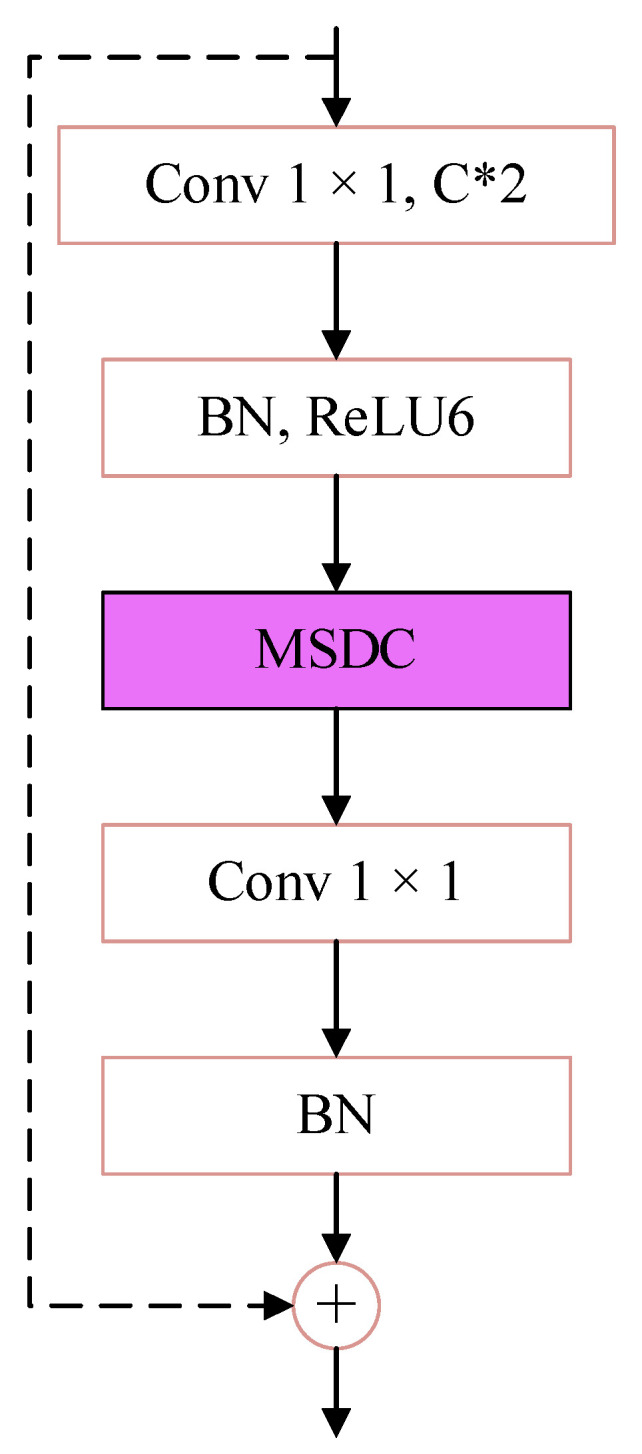
Architecture of the multi-scale convolution block (MSCB) with channel shuffle and residual connections for efficient multi-scale feature fusion.

In YOLO-Night, C3k2-MSCB is integrated into both the backbone and neck to strengthen multi-scale feature extraction under adverse illumination, contributing to improved detection performance for small and partially visible objects in dark environments. The structures of the MSDC and MSCB modules are shown in Figure 6 and Figure 7, respectively.

### 3.5. Improved Detection Head

Unlike daytime scenes, nighttime and low-light driving scenes are dominated by small, distant, and low-contrast objects whose visual cues are easily suppressed during deep feature extraction. In the original YOLOv11 architecture, object detection is performed on three feature scales (P3–P5), which limits the preservation of fine-grained spatial details necessary for reliable detection under adverse illumination.

**Figure 8:**
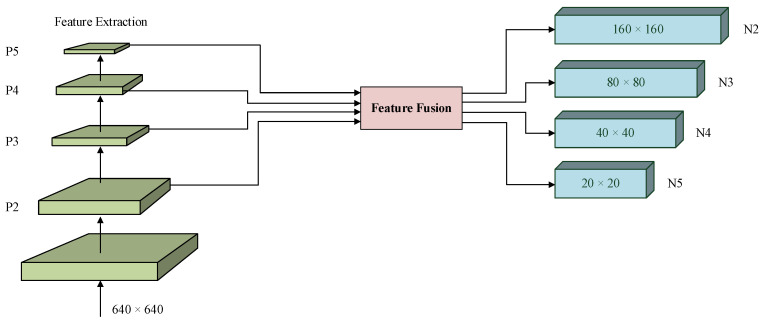
Detection head structure of YOLO-Night with the additional P2 branch, enabling enhanced sensitivity to small and low-contrast objects in nighttime scenes.

To enhance small-object sensitivity, YOLO-Night introduces an additional P2 detection head, as illustrated in Figure 8.

This design enables detection on four feature scales corresponding to downsampling factors of 4×, 8×, 16×, and 32×, allowing the network to better retain spatial information critical for micro and small targets in dark environments. The configuration of the improved detection heads is summarized in Table 1.

**Figure 9:**
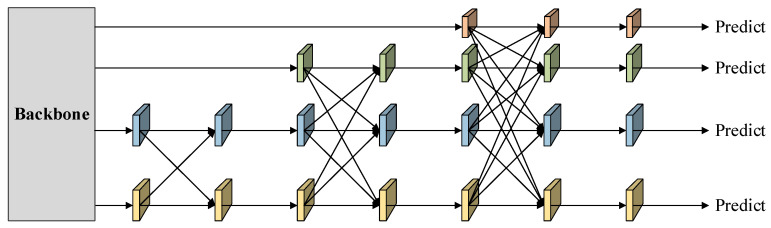
Architecture of the Asymptotic Feature Pyramid Network (AFPN) with staged feature fusion and adaptive spatial weighting for multi-scale alignment.

To further improve cross-scale feature fusion, the Asymptotic Feature Pyramid Network (AFPN) [13] was employed in the detection head. AFPN performs staged feature fusion to progressively align semantic information across different feature levels, reducing the semantic gap commonly introduced by direct cross-layer fusion. In addition, an adaptive spatial feature fusion (ASFF) mechanism [35] was incorporated to dynamically balance spatial contributions from different scales, enabling the more precise localization of low-contrast targets. The overall AFPN structure is shown in Figure 9.

By combining a low-level detection branch with staged multi-scale feature fusion, the improved detection head enhances robustness to scale variation and contrast degradation in nighttime object detection.

## 4. Experimental Verification

### 4.1. Dataset

All experiments in this study were conducted on the NightCity dataset [36], a large-scale nighttime semantic segmentation dataset designed for autonomous driving scenarios. NightCity contains 2998 training images and 1299 validation images, each with a resolution of 1024 × 512, collected across multiple countries and diverse nighttime lighting conditions, including dimly lit urban environments, shaded regions, and low-visibility scenes.

To enable object detection evaluation, the NightCity dataset was converted into a bounding-box-based detection format compatible with the YOLO framework. Specifically, object instances were derived from semantic annotations and transformed into rectangular bounding boxes enclosing each labeled region. Following conversion, the dataset was divided into training, validation, and testing subsets using a 6:2:2 ratio. This split was applied consistently across all evaluated methods to ensure fair comparison.

Although random splitting was adopted in this study, we note that scene-level correlations may exist in nighttime datasets. The impact of dataset partitioning on performance is discussed as a limitation in the future work section.

### 4.2. Experimental Environment

The experimental setup used in this study used a workstation equipped with an Intel^®^ Core™ i7-12700K CPU (Intel, Santa Clara, CA, USA) and an NVIDIA GeForce RTX 3090 GPU (NVIDIA, Santa Clara, CA, USA) with 24 GB of video memory. The system was configured with 32 GB of RAM and operated under Windows 11. The implementation was based on Python 3.10, with CUDA and cuDNN libraries used to accelerate model training and inference. Detailed hardware and software configurations are summarized in Table 2. All comparative experiments were performed under identical settings to ensure reproducibility and fairness.

### 4.3. Evaluation Metrics

To evaluate detection performance, precision (P), recall (R), and mean average precision (mAP) were employed in this study. Model efficiency was assessed using the number of floating-point operations (FLOPs) and the total parameter count. Precision and recall measure the accuracy of positive predictions and the ability to detect ground-truth objects, respectively, and are defined as:

(8) $$Precison=\frac{TP}{TP+FP}$$

(9) $$Recall=\frac{TP}{TP+FN}$$

where TP (True Positive) represents the number of correctly identified samples, FP (False Positive) represents the number of incorrectly identified positive classes, and FN (False Negative) represents the number of samples that the model failed to recognize.

Average precision (AP) is computed for each object category based on the precision–recall curve, and the mean average precision (mAP) is obtained by averaging AP across all categories:

(10) $$AP=\int_{0}^{1}P(R)dR$$

(11) $$mAP=\frac{\sum_{i=1}^{N}AP_{i}}{N}$$

where N represents the total number of object categories. mAP is reported at an intersection-over-union (IoU) threshold of 0.5 (mAP@50). FLOPs are computed based on an input resolution of 640 × 640 to reflect inference complexity, while parameter count indicates the model size.

### 4.4. Ablation Experiment

To evaluate the contribution of individual components in YOLO-Night, ablation experiments were conducted by incrementally introducing each module into the YOLO11n baseline. The results are summarized in Table 3, where P indicates that the corresponding modules in the baseline model have been modified.

Table 3 shows that introducing FFA-Net led to a moderate improvement in recall (+1.9%) and mAP@50 (+0.6%), indicating enhanced sensitivity to low-contrast targets in nighttime scenes. This improvement was accompanied by an increase in computational cost, reflecting the additional feature conditioning overhead introduced at the early stage. The inclusion of DSAC improved precision (+3.1%) and mAP@50 (+1.5%) while maintaining comparable computational complexity. This suggests that adaptive receptive field modeling enhances localization accuracy for blurred and multi-scale targets without incurring significant efficiency penalties.

When the C3k2-MSCB module was enabled, recall increased notably (+3.1%), demonstrating improved multi-scale feature preservation under low-light conditions. Importantly, this gain was achieved with only a marginal increase in FLOPs, indicating a favorable balance between performance and efficiency. The addition of the P2-AFPN detection head further improved the precision and mAP@50 by strengthening small-object detection and cross-scale feature alignment. Although this introduced additional computation, the increase remained moderate relative to the overall performance gain.

Finally, combining all proposed modules yielded the best overall performance, achieving improvements of +14.3% in precision, +7.6% in recall, and +5.7% in mAP@50 compared to the baseline. These results demonstrate that the proposed components are complementary rather than redundant, and their joint integration leads to a balanced improvement in detection accuracy and robustness under nighttime conditions.

### 4.5. Comparative Experiments

To evaluate the effectiveness of the proposed YOLO-Night framework, comparative experiments were conducted against different real-time object detection models on the NightCity dataset. The comparison focused on detection accuracy, robustness under nighttime conditions, and computational efficiency. All baseline models were initialized using their officially released pretrained weights and subsequently fine-tuned on the NightCity dataset using a unified training protocol. Specifically, all methods were trained with the same optimizer, learning rate schedule, batch size, number of epochs, and data augmentation strategy. This ensures that performance differences among the compared methods arise from architectural design choices rather than differences in training strategies or dataset adaptation. The quantitative results of comparison are summarized in Table 4.

As shown in Table 4, early lightweight YOLO variants such as YOLOv3-tiny, YOLOv5n, YOLOv8n, and YOLOv10n, exhibit limited robustness under nighttime conditions, particularly in recall and mAP, indicating their sensitivity to low-contrast and low-illumination environments. Nighttime-specific methods such as Dark-YOLO, LOL-YOLO, and 3L-YOLO improve detection accuracy by incorporating enhancement or multi-scale strategies; however, these gains are often accompanied by increased computational cost or unstable performance under extreme darkness.

More complex models, including RT-DETR, NLE-YOLO, and DimNet, achieve higher mAP values by leveraging transformer-based architectures and frequency-domain feature enhancement. While these approaches demonstrate strong detection capability, they incur substantial computational overhead, with FLOPs exceeding 100 × 10^9^ in some cases, limiting their suitability for real-time deployment in autonomous driving systems.

In contrast, YOLO-Night achieved the highest precision among all compared methods, reaching 86.6% while maintaining competitive recall and mAP performance with significantly lower computational complexity. Although its mAP@50 was slightly lower than that of the most computationally intensive models, YOLO-Night required only 8.5 × 10^9^ FLOPs and 3.8 M parameters, enabling real-time inference without sacrificing detection reliability. This balance between high precision, robust recall, and moderate computational cost demonstrates that YOLO-Night effectively addresses the trade-off between accuracy and efficiency, making it well-suited for practical nighttime autonomous driving applications.

To further analyze the generalization capability of YOLO-Night across different object categories, we conducted a category-wise performance comparison against representative YOLO models. Detection results for 6 object categories in the NightCity dataset are reported in Table 5 using mAP@50 as the evaluation metric.

As shown in Table 5, YOLO-Night achieved the highest detection accuracy in all 6 categories. Significant improvements were observed for small and low-contrast objects such as car, bicycle, and motorbike, where YOLO-Night outperformed the YOLO11n baseline by 10.3%, 2.4%, and 10.2%, respectively. These results indicate that the proposed framework enhances robustness not only at the aggregate level but also across diverse object categories commonly encountered in nighttime driving scenarios.

### 4.6. Visual Analysis

**Figure 10:**
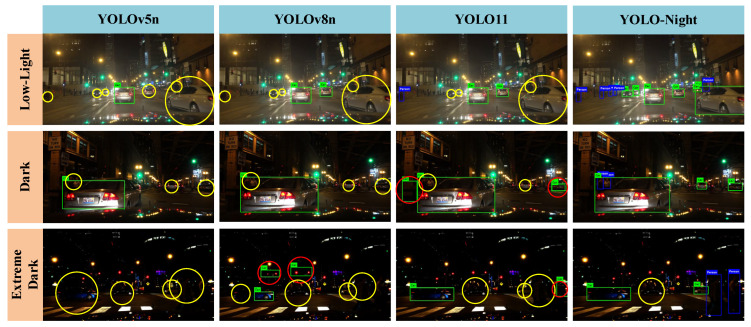
Visual comparison of detection results produced by YOLOv5n, YOLOv8n, YOLO11n, and YOLO-Night under low-light, dark, and extremely dark nighttime scenarios. Yellow circles indicate missed detections, and red circles denote false detections.

To qualitatively evaluate detection performance under nighttime conditions, YOLO-Night was tested on the NightCity test set as well as additional real-world nighttime images. Representative detection results were selected for visualization to provide an intuitive comparison between the baseline YOLO11n and the proposed YOLO-Night framework. Figure 10 presents qualitative comparisons among YOLOv5n, YOLOv8n, YOLO11n, and YOLO-Night, where missed detections are highlighted with yellow circles and false detections are marked with red circles.

As shown in Figure 10, the baseline YOLO11n model was able to detect large and nearby objects under mildly low-light conditions; however, its performance degraded noticeably in darker scenes, particularly in the presence of strong reflections, low contrast, or extremely dark backgrounds.

From the visual comparisons in Figure 10, several observations can be made:

1. Low-light scenarios with reduced contrast.

YOLO-Night demonstrated more reliable detection results, with fewer missed and false detections compared to baseline models. In these scenes, YOLO-Night preserved object boundaries more effectively and improved the detection of small-scale targets that are often overlooked by other models.

2. Dark environments with blurred targets.

YOLO-Night showed improved robustness to motion blur and illumination degradation, enabling more accurate detection of both blurred and small objects. In contrast, YOLOv5n, YOLOv8n, and YOLO11n frequently missed small targets or generated spurious detections under similar conditions.

3. Extremely dark scenarios.

In extreme low-illumination environments, baseline YOLO models failed to detect most objects, and YOLOv8n occasionally produced false detections, such as misclassifying light sources as vehicle rear lights. YOLO-Night substantially reduced such errors, with remaining missed detections primarily occurring when object appearance closely blended with the background.

The visual results in Figure 10 corroborate the quantitative findings reported in Section 4.5. YOLO-Night consistently improved detection robustness across low-light, dark, and extremely dark scenarios, particularly for small, blurred, and low-contrast targets. These qualitative results further demonstrate the practicality and generalization capability of the proposed framework for nighttime object detection in autonomous driving applications.

## 5. Conclusions

This work addresses the challenge of degraded perception performance in autonomous vehicles operating under nighttime and low-illumination conditions. We proposed YOLO-Night, a detection framework that enhances robustness to low contrast, blur, and small objects through integrated architectural adaptations while maintaining real-time efficiency. Experimental results on the NightCity dataset demonstrated that YOLO-Night achieved a 14.3% improvement in precision and a 10.4% increase in mAP@50 over the YOLO11n baseline, reaching 86.6% precision and 72.7% mAP@50. Compared with widely used lightweight detectors such as YOLOv5n, YOLOv8n, and YOLOv10n, YOLO-Night consistently delivered superior detection performance under nighttime conditions with a moderate and controllable increase in computational cost. Qualitative visual analyses further corroborate these findings, showing improved robustness in detecting low-contrast regions, blurred objects, and small targets across diverse nighttime scenarios. Despite these improvements, detection performance remains challenging in extremely dark environments where object appearance closely blends with the background. In such cases, YOLO-Night exhibited a residual miss-detection rate of approximately 2%, indicating that extreme illumination degradation remains an open problem.

Future work will focus on further optimizing network efficiency, enhancing robustness under extreme low-light conditions, and exploring adaptive or multimodal perception strategies to improve reliability without compromising real-time performance. Extending the proposed framework to broader autonomous perception tasks may also contribute to advancing practical applications in Advanced Driver Assistance Systems (ADAS) and other safety-critical domains.

## Figures and Tables

**Table 1:** Configuration of multi-scale detection heads in YOLO-Night, including feature map resolution and corresponding target size ranges.

Name	Feature Map Size	Detection Target Size	Detection Size
P2/4	160 × 160	4 × 4 or larger	Micro targets
P3/8	80 × 80	8 × 8 or larger	Small targets
P4/16	40 × 40	16 × 16 or larger	Medium targets
P5/32	20 × 20	32 × 32 or larger	Large targets

**Table 2:** Hardware and software configuration used for training and evaluation of all models.

Configuration	Name	Specific Information
Hardware	CPU	Intel (R) Core i7-12700K
	GPU	NVIDIA GeForce RTX 3090
	VRAM	24 G
	RAM	32 G
Software	Operating system	Windows 11
	Python	3.10
	CUDA	13.0
	cuDNN	9.16.0

**Table 3:** Ablation study results on the NightCity dataset. Precision (P) and Recall (R) are reported at IoU = 0.5 using a confidence threshold of 0.25 after non-maximum suppression, following the standard YOLO evaluation protocol. mAP@50 and mAP@50:95 are computed by integrating over confidence thresholds.

YOLO11n	FFA-Net	DSAC	MSCB	P2-AFPN	P %	R %	mAP@50%	mAP@95%	FLOPs/10^9^
✓	×	×	×	×	72.3	56.9	62.3	39.0	6.5
✓	✓	×	×	×	74.1	58.8	62.9	39.4	7.1
✓	×	✓	×	×	75.4	56.2	62.8	38.0	6.4
✓	×	×	✓	×	73.9	60.0	63.1	38.9	6.7
✓	×	×	×	✓	74.7	55.6	62.3	39.2	7.4
✓	✓	✓	×	×	80.1	60.0	62.7	40.6	7.7
✓	✓	✓	✓	×	84.9	63.5	67.3	40.7	8.2
✓	✓	✓	✓	✓	86.6	69.3	72.7	49.1	8.5

**Table 4:** Unified comparison of YOLO-based and nighttime-oriented object detection models on the NightCity dataset. For detection metrics, higher values indicate better performance. For efficiency metrics (FLOPs and parameter count), lower values indicate better efficiency.

Model	P %	R %	mAP@50%	mAP@50:95%	FLOPs/10^9^	Params/10^6^
YOLOv3-tiny	68.0	51.3	56.5	30.8	14.3	9.5
YOLOv5n	70.6	52.3	60.4	35.6	**5.8**	**2.2**
Dark-YOLO	70.6	52.3	62.2	37.2	7.5	2.9
YOLOv8n	71.3	56.9	61.7	37.1	7.0	2.7
YOLOv10n	71.9	52.8	58.6	35.7	8.2	3.1
YOLO11n	72.3	56.9	62.3	42.5	6.3	2.6
RT-DETR	75.3	60.0	69.6	42.6	103.5	32.0
3L-YOLO	76.0	59.3	68.8	42.0	8.1	5.9
LOL-YOLO	70.9	62.5	68.1	42.3	20.6	5.7
NLE-YOLO	78.2	64.5	71.3	43.4	221.2	3.4
DimNet	79.1	67.7	75.6	48.0	26.7	5.4
YOLO-Night (Ours)	86.6	69.3	72.7	49.1	8.5	3.8

**Table 5:** Comparison of different model performance by category (mAP@50).

Model	YOLOv5n	YOLOv8n	YOLOv10n	YOLO11n	Proposed Model
All	60.4	61.7	58.6	62.3	72.7
Bicycle	71.8	73.2	68.5	73.0	75.4
Bus	81.6	80.5	80.6	82.3	87.2
Car	73.0	71.9	68.6	73.1	83.4
Motorbike	64.2	67.3	60.2	60.0	70.2
Person	68.5	69.4	64.2	71.9	78.2
Truck	49.2	49.3	48.9	52.7	68.1

## Data Availability

The data presented in this study are available on request from the corresponding author due to privacy and legal reasons.
